# Evaluation of Predictors of Biochemical Recurrence in Prostate Cancer Patients, as Detected by ^68^Ga-PSMA PET/CT

**DOI:** 10.3390/diagnostics12010195

**Published:** 2022-01-14

**Authors:** Mads T. Christensen, Mads R. Jochumsen, Søren Klingenberg, Karina D. Sørensen, Michael Borre, Kirsten Bouchelouche

**Affiliations:** 1 Department of Nuclear Medicine, PET-Centre, Aarhus University Hospital, 8200 Aarhus, Denmark; madsjoch@rm.dk (M.R.J.); SOEKLI@rm.dk (S.K.); Kirsten.Bouchelouche@auh.rm.dk (K.B.); 2 Department of Clinical Medicine, Aarhus University, 8000 Aarhus, Denmark; kdso@clin.au.dk (K.D.S.); borre@clin.au.dk (M.B.); 3 Department of Molecular Medicine, Aarhus University Hospital, 8200 Aarhus, Denmark; 4 Department of Urology, Aarhus University Hospital, 8200 Aarhus, Denmark

**Keywords:** prostate cancer, ^68^Ga-Prostate-Specific Membrane Antigen PET/CT, ^68^Ga-PSMA, biochemical recurrence, detection rate, predictors

## Abstract

Objectives: To explore the existence of new predictors of the ^68^Ga-Prostate-Specific Membrane Antigen (PSMA) PET/CT detection rate at biochemical recurrence (BCR) and to determine the detection rate of ^68^Ga-PSMA PET/CT dependent of prostate-specific antigen (PSA) levels. Materials and methods: In total, 189 PCa patients scanned with ^68^Ga-PSMA PET/CT for detection of BCR after curatively intended treatment with either radical prostatectomy (*n* = 153) or radiotherapy (*n* = 36) were included. Clinicopathological information at the time of diagnosis (PSA, clinical tumor-stage, International Society of Urological Pathology Grade Group and whether ^68^Ga-PSMA PET/CT was used for primary staging), treatment (RT/RP and histopathology of the prostatectomies), and pre-PET PSA were collected from medical records. Results: Of the 189 ^68^Ga-PSMA PET/CT scans, 103 (54.5%) were positive for BCR of PCa. No significant coherency was observed between detection rate and any clinicopathological variables at diagnosis. Detection rates significantly increased with rising PSA: <0.5 ng/mL = 28%, 0.5 ≤ 1 ng/mL = 39%, 1 ≤ 2 ng/mL = 64%, 2 ≤ 5 ng/mL = 87.5% and ≥5 ng/mL = 97%. Conclusions: The detection rate of PCa recurrence was strongly dependent of pre-PET PSA levels. None of the additional clinical variables acquired during primary staging, prostatectomy pathology reports, nor primary staging imaging modality affected the detection rate.

## 1. Introduction

Prostate cancer (PCa) is the second most commonly diagnosed male cancer, accounting for 358.989 registered cases of death in 2018, and thereby being the fifth leading cause of cancer death among men worldwide [1]. The options for curative intended treatment of PCa are either radical prostatectomy (RP) or radiotherapy (RT). As a result of different treatment options for the primary disease, biochemical recurrence (BCR) is, according to the standard care in Denmark, defined by PSA ≥ 0.2 ng/mL post RP and by PSA reaching nadir + 2.0 ng/mL post RT [2]. Relapse detection of BCR has become a well-established indication for ^68^Ga-Prostate-Specific Membrane Antigen (PSMA)-PET/CT [3]. Several PSMA ligands have been developed, with ^68^Ga-PSMA-11(Glu-CO-Lys(Ahx)-HBED-CC) being one of the most commonly used compounds [4].

^68^Ga-PSMA PET/CT is a commonly used modality in patients with BCR, as it has been shown to be superior to conventional diagnostic imaging in locating recurrent disease [5,6,7,8]. This advantage is especially noticeable in cases with low PSA values (<0.5 ng/mL) [9,10]. In the case of BCR, it is crucial to locate the anatomical site of recurrence regarding treatment options where ^68^Ga-PSMA PET/CT has shown to influence the management of patients with recurrent PCa [11]. While an association has been found between increasing PSA levels and the detection rate of ^68^Ga-PSMA PET/CT in recurrent disease, most studies did not report clinical data from the time of diagnosis, and hence no additional predictors of detection rate have been described. In the present cohort, we have access to imaging and clinical data from both the primary staging, treatment and BCR.

Hence, the aim of this retrospective study was to investigate whether any of these reported clinicopathological variables could serve as possible predictors of the detection rate of ^68^Ga-PSMA PET/CT in patients with recurrent disease. An additional aim of the study was to compare the ^68^Ga-PSMA PET/CT detection rates dependent on pre-PET PSA levels with results from the existing literature.

## 2. Materials and Methods

### 2.1. Patient Population

**Figure 1:**
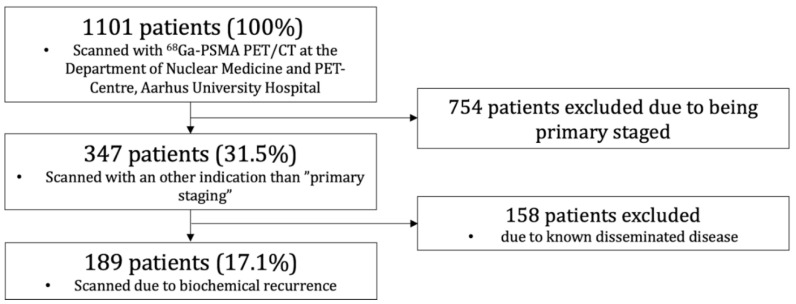
Study flow for ^68^Ga-PSMA PET/CT of patients with biochemical recurrence.

From April 2016 through March 2019, a total of 1101 ^68^Ga-PSMA PET/CT scans were conducted at the Department of Nuclear Medicine and PET-Centre, Aarhus University Hospital, Denmark. PCa patients scanned with ^68^Ga-PSMA PET/CT for detection of BCR after curatively intended treatment with either RP or RT were included. Patients referred for primary staging or with previously proven dissemination were excluded. Of the 1101 patients, a total of 189 (17.1%) met the criteria (Figure 1).

Data was available through medical records and collected and managed using REDCap electronic data capture tools, hosted at Aarhus University [12,13]. Clinicopathological information at the time of diagnosis (PSA (latest before treatment), clinical tumor (cT)-stage, Gleason Score, International Society of Urological Pathology (ISUP) Grade Group [14], and whether ^68^Ga-PSMA PET/CT was used for primary staging), information concerning their treatment (RP/RT and pathology in case of RP) and lastly, clinical information at the time of recurrent disease (PSA at scan, results and location of recurrent disease) were attained. The ISUP Grade Group is defined as; Group 1 = Gleason Score 6, Group 2 = Gleason Score 7 (3 + 4), Group 3 = Gleason Score 7 (4 + 3), Group 4 = Gleason Score 8, and Group 5 = Gleason Score 9–10 [14]. As some patients were diagnosed before the establishment of the electronical medical records and some were referred from other regions of Denmark (thus resulting in inaccessible records), information regarding certain variables, especially about the time of diagnosis, was unavailable in some patients. The time from primary diagnosis to first diagnostic imaging varies greatly in this cohort, as some patients are examined in fast track while low risk patients could have gone in active surveillance for years before the first diagnostic imaging.

This retrospective study was approved by the institutional review board (Central Denmark Region Committees on Health Research Ethics).

### 2.2. ^68^Ga-PSMA PET/CT

The production of ^68^Ga-PSMA-11 (^68^Ga-Glu-CO-Lys(Ahx)-HBED-CC) was produced according to good manufacturing practice (GMP) and QC following the national Danish regulations. All ^68^Ga-PSMA PET/CT scans were performed according to EANM and SNMMI guidelines [15]. The patients did not fast before the scan, but were well hydrated before the study and during the tracer uptake time (e.g., oral intake of 500 mL of water during a 2 h period prior to acquisition). The patients received an intravenous bolus injection of 2.14 MBq ^68^Ga-PSMA-11 per kilogram bodyweight approximately 60 min before the scan. The injection of the tracer was followed by flushing of the syringe with at least the same volume of saline (NaCl 0.9%) to maximize use of dispensed activity. ^68^Ga-PSMA PET/CT scans were performed with low-dose CT for anatomical localization and attenuation correction. Just before the static PET/CT scan, the patients emptied their urinary bladder. All scans were performed with a Siemens Biograph TruePoint PET/CT scanner (Siemens, Erlangen, Germany). The patients were positioned with both arms elevated above the head, as tolerated by the patients. The patients were PET/CT scanned from vertex cranii to mid-thigh. All available corrections (attenuation, scatter and Point-Spread Function) were applied in the image reconstruction using the TrueX reconstruction algorithm (4 iterations and 21 subsets) and a 3 mm Gaussian post-filter (XYZ) and voxel size of 2 × 2 × 2 mm.

### 2.3. Image Analysis

The analyses of all scans were performed by experienced, board certified specialists in nuclear medicine, in most cases by a single reader with many years of experience in prostate cancer PET imaging. A second evaluation was performed by an experienced specialist at the weekly prostate cancer multidisciplinary team conference. In cases of disagreement, a consensus was made between the experienced specialists. Image analysis was performed using Hybrid Viewer (HERMES Medical Solutions AB, Stockholm, Sweden). The scan results were defined as “positive”, “negative” or “equivocal”. Positive was defined as visually avid focal tracer uptake not associated with physiological uptake and higher than adjacent tissue, and hence suspicious of malignancy, these criteria are in accordance with Prostate Cancer Molecular Imaging Standardized Evaluation (PROMISE) guidelines [16] and with the major publications on this topic [15,17,18]. Equivocal was defined as cases with increased focal tracer uptake where it was not possible to distinguished between a benign or malignant condition, which in most cases leads to a follow-up biopsy or MRI scan. Negative was defined as no suspicious tracer uptake. Pathological ^68^Ga-PSMA uptake in the prostatic bed was defined as “local”, uptake in pelvic lymph nodes below the common iliac artery bifurcation was defined as lymph node metastases (LNMs), and thus “N1” disease (PROMISE-criteria [16]), whereas extra pelvic LNMs were defined as “M1a”. Lesions proposing bone metastases and visceral metastases were defined as “M1b” and “M1c”, respectively.

The suspicious lesions on ^68^Ga-PSMAPET/CT were biopsied, when possible and clinically relevant, for histopathological confirmation of the findings as reference standard. The involved specialists were aware of the possible pitfalls when reading the PSMA PET/CT scans [19].

### 2.4. Statistical Analysis

Statistical analyses were performed using Stata 16.1. The variables were summarized using descriptive statistics. Continuous variables were examined for normal distribution using Q-Q plots. If the continuous variables were not normally distributed, they were presented as median (range). Detection rate was defined as the proportion of patients with positive ^68^Ga-PSMA PET/CT scans in comparison to patients with negative or equivocal results. Equivocal results were grouped with the negative scans due to their uncertainty and inability to distinguish between benign and malignant lesions, thereby not being able to define the lesions as “positive”. Detection rates were compared in different analyses with stratification for different variables using χ^2^-tests with a significance level of *p* < 0.05 using the actual numbers for positive and negative scans.

## 3. Results

### 3.1. Patient Summary

Of the 189 ^68^Ga-PSMA PET/CT recurrence scans, a total of 103 (54.5%) were positive, 12 (6.3%) were equivocal and grouped with the 74 (39.2%) negative scans. Therefore, a total of 86 (45.5%) scans were defined as negative in the analysis.

Regarding treatment prior to the ^68^Ga-PSMA PET/CT scan, 153 (81%) underwent RP and 36 (19%) underwent RT with a detection rate of 44.4% and 97.2% in the RP and RT groups, respectively (Table 1).

### 3.2. ^68^Ga-PSMA PET/CT Detection Rate and Tumor Characteristics during Primary Staging

PET detection rate of the relapse was compared when stratifying for different variables acquired during primary staging (Table 2). Analyses were performed when taking the latest PSA before treatment (<10, 10–20, >20 ng/mL), cT-stage (≤cT2a, cT2b, ≥cT2c), ISUP Grade Group and risk stratification (D’Amico classification [20]) individually into consideration. None of these variables affected the PET detection rate (Table 2) (*p* > 0.05).

### 3.3. ^68^Ga-PSMA PET/CT Detection Rate in Patients Treated with RP

In the 153 patients treated with RP, information regarding ISUP Grade Group, surgical margins and pathological tumor (pT)-stage was accessible in most patients (Table 3). The detection rate was independent of these pathological characteristics (*p* > 0.05).

Recategorizing pT-stage (<pT3a and ≥pT3a) and ISUP Grade Group (<4 and ≥4) did not affect the results (*p* > 0.05).

### 3.4. Primary Staged Using ^68^Ga-PSMA PET/CT vs. Other

Forty patients (21.2%) had ^68^Ga-PSMA PET/CT conducted for primary staging purposes as well as having ^68^Ga-PSMA PET/CT conducted due to BCR. The rest of the patients (*n* = 149 (78.8%)) had a ^18^F-choline-PET, bone scintigraphy and/or CT conducted or no imaging during their primary staging.

The detection rate was substantially lower for the patients with ^68^Ga-PSMA PET/CT for primary staging purposes (35%) compared to patients with other or no imaging for primary staging (59.7%). The patients with ^68^Ga-PSMA PET/CT staging had lower PSA levels as well, and after stratification for PSA, the impact of imaging modality for primary staging disappears.

### 3.5. PSA-Level at Recurrent Disease

**Figure 2:**
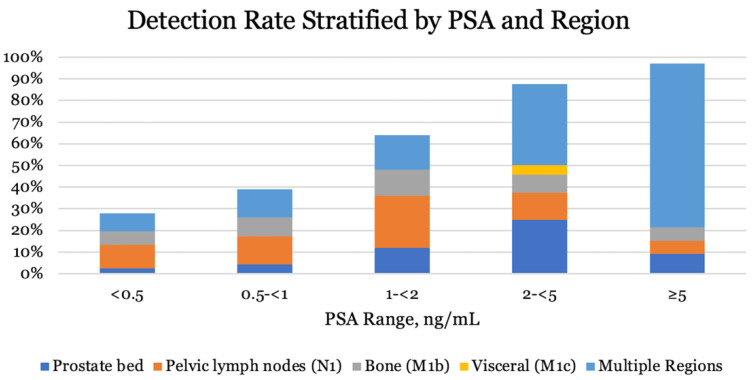
Proportion of patients with ^68^Ga-PSMA PET/CT positive findings, stratified by PSA and region in all patients (*n* = 189).

PSA right before ^68^Ga-PSMA PET/CT at BCR was available in 187 patients (98.9%). Median PSA at BCR was 0.62 ng/mL (0.1–127) for all 187 patients, whereas median PSA was 0.4 ng/mL (0.1–127) in the RP group and 4.9 ng/mL (0.1–124) in the RT group. Detection rate stratified by PSA is given in Table 4. A significant increase in detection rate was observed for all PSA intervals. Similar results were observed in the RP group, only with a small decrease in the detection rate (Table 5) compared to the joint patient group (RP + RT). Not only an increase in detection rate was observed, disease was more likely to spread to multiple locations with increasing PSA as well (Figure 2).

Analysis including RT patients only was not conducted due to the few numbers of patients and due to the fact that nearly all RT patients were in the 2 ≤ 5 or ≥5 ng/mL strata.

## 4. Discussion

The use of ^68^Ga-PSMA PET/CT in PCa has shown great potential in the clinical management of PCa, both for primary staging of high risk PCa and for detection of biochemical recurrence [21]. PSMA expression has been shown to be amplified in PCa with high preoperative PSA, tumor Gleason Score and advanced tumor stage [22]. In accordance with this, maximum standardized uptake value (SUV_max_) has been shown to correlate with ISUP Grade Group in the primary tumor [23,24,25]. Regarding recurrent disease, Vinsensia et al. [26] (*n* = 147) demonstrated a higher SUV_max_ in LNMs with high Gleason Scores (≥8) than in intermediate and low grade PCa. While an association has been found between increasing PSA levels and the detection rate of ^68^Ga-PSMA PET/CT in recurrent disease, most studies did not report clinical data from the time of diagnosis, and hence no additional predictors of detection rate have been described.

The aim of this retrospective study was to investigate whether several variables like clinicopathological information at the time of diagnosis (PSA, clinical tumor-stage, International Society of Urological Pathology Grade Group and whether ^68^Ga-PSMA PET/CT was used for primary staging), treatment (RT/RP and histopathology of the prostatectomies), and pre-PET PSA could serve as possible predictors of the detection rate of ^68^Ga-PSMA PET/CT in patients with recurrent disease. The study showed no significant association between the detection rate of ^68^Ga-PSMA PET/CT at the time of recurrent disease and characteristics of the primary cancer at baseline or at RP. Similarly, having ^68^Ga-PSMA PET/CT conducted for primary staging did not seem to affect the detection rate of ^68^Ga-PSMA PET/CT in BCR, when compared to primary staging with conventional imaging (bone scan and CT). Recently, a large study compared ^68^Ga-PSMA PET/CT with CT and bone scan in primary staging of high risk PCa [27]. The study showed that ^68^Ga-PSMA PET/CT was superior as compared to conventional imaging, with a higher overall accuracy (92% vs. 65%), sensitivity (85% vs. 38%) and specificity (98% vs. 91%). Furthermore, ^68^GaPSMA PET/CT also led to more frequent changes in treatment plan. In our study, we investigated if the imaging modality used for primary staging affected the detection rate of ^68^Ga-PSMA PET/CT at biochemical recurrence. Though different detection rates were observed, the median PSA of the patients with ^68^Ga-PSMA PET/CT for primary staging was 0.3 ng/mL compared with 1 ng/mL in the group of patients without ^68^Ga-PSMA PET/CT for primary staging (staging with other imaging modalities like bone scan and CT). While imaging modality for primary staging at first seemed to influence the detection rate of ^68^Ga-PSMA PET/CT in recurrent disease, the impact disappeared when stratifying for PSA. The non-balanced group sizes could have influenced the findings, but it is important to underline that this observation says nothing about the performance of ^68^Ga-PSMA PET/CT vs. conventional imaging as primary staging modality. Hence, the detection rate seemed to be independent of any primary staging and perioperative characteristics, resulting in no new predictors of the ^68^Ga-PSMA PET/CT detection rate being identified.

**Figure 3:**
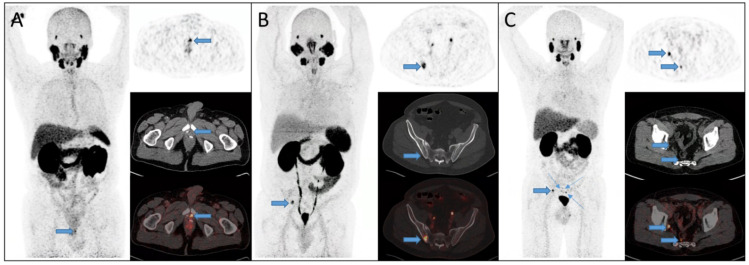
Patients with detected relapse on ^68^Ga-PSMA PET/CT. (**A**) A 75 year-old man with biochemical recurrence (PSA = 0.5 ng/mL) seven years after radical prostatectomy. Local relapse was visualized on ^68^Ga-PSMA PET/CT just anterior to the surgical clips (arrows). Subsequently, radiation therapy was performed on the relapse, and now four years later the patient has unmeasurably low PSA. (**B**) A 67 year-old male with biochemical recurrence (PSA = 5 ng/mL) after salvage radiation therapy. A bone metastasis in the right iliac bone was found (arrows). Subsequently the patient underwent stereotactic radiation therapy of the metastasis with currently two years free of progression. (**C**) A 59 year-old man with biochemical recurrence (PSA = 1.6 ng/mL) three years after radical prostatectomy. ^68^Ga-PSMA PET/CT showed small pelvic and mesorectal lymph node metastases (arrows).

In agreement with multiple other studies [9,17,28,29], we found that the detection rate was highly associated with increasing pre-PET PSA. In patients with PSA levels below 1 ng/mL, the relapse was only detected in approximately one-third of the patients, whereas the detection rate approached 100% at PSA levels above 5 ng/mL. As seen in Figure 2, increasing PSA did not only affect the detection rate, but also increased the frequency of dissemination to multiple locations as well. These findings are in agreement with the findings by Fendler et al. [17]. The low detection rate at low PSA levels is probably explained by the lower limit of detection for ^68^Ga-PSMA PET/CT, meaning that micro metastatic deposits of PSMA expressing cells remain undetected by ^68^Ga-PSMA PET/CT [23]. Patient cases of detected relapse are provided in Figure 3.

The detection rates from this study seemed slightly lower than that of other studies. Yet, a large variation between different studies can be observed in the low PSA range [9]. In this study, the equivocal scans (*n* = 12) were pooled with the patients with negative scans, thus perhaps underestimating the detection rate. It is also worth noticing that subdividing the <0.5 ng/mL stratum into 0–0.19 and 0.20–0.49 ng/mL had an impact on the detection rate (33% and 45%, respectively) [9], which is why differences in mean PSA in this stratum can affect the detection rate between studies.

The detection rate in this study was 98.8% in the RT patients and thereby markedly higher than in the RP patients (44.4%). However, due to differences in the definition of BCR, patients treated with RT by definition have PSA levels larger than that of patients treated with RP (median 4.9 ng/mL and 0.4 ng/mL, RT and RP, respectively). The difference in the detection rate can for that reason presumably be ascribed to these differences in PSA.

The retrospective nature represents a primary limitation to the current study, as it can imply potential bias. Additionally, the lack of histopathological verification of all PSMA avid lesions on PET provides uncertainty regarding the results of the scans. The relatively small sample size may affect the lack of significance in the association of several of the tested characteristics and the detection rate of ^68^Ga-PSMA PET/CT.

Androgen deprivation therapy (ADT) as a possible predictor of the detection rate has been discussed [28,30]. Preclinical and clinical data indicate that PSMA expression is increased during ADT [31], especially shortly after initiation of ADT, but the clinical impact of ADT on ^68^GA-PSMA PET/CT performance requires further study, as mentioned in the guidelines [15]. In our study, information regarding ADT as well as other kinds of treatment between RP or RT and ^68^Ga-PSMA PET/CT for recurrent disease was unavailable in some medical records. Thus, information on ADT was not included in the study, and no sub-analysis on ADT was performed. However, we did not expect that the ^68^Ga-PSMA PET/CT scans were performed shortly after the beginning of possible ADT. It is well-known that PSA velocity and PSA doubling time are correlated to ^68^Ga-PSMA PET/CT detection rate at biochemical recurrence, however, information regarding PSA velocity and PSA doubling time has not been taken into account in this study due to unavailability in several patients.

## 5. Conclusions

Of 189 PCa patients with biochemical recurrence, the relapse was detected on ^68^Ga-PSMA PET/CT in 103 patients (54.5%). No new predictors of the ^68^Ga-PSMA PET/CT detection rate were identified, as none of the clinical variables acquired during primary staging, pathology reports from prostatectomies, nor imaging modality during primary staging affected the detection rate of ^68^Ga-PSMA PET/CT.

The detection rate of ^68^Ga-PSMA PET/CT was strongly dependent on PSA at recurrence (*p* < 0.05) with increased detection rates, with increasing PSA in agreement with existent literature.

## Figures and Tables

**Table 1:** Patient summary (All patients).

Characteristic	All Patients, *n* = 189	Positive Scans, *n* = 103	Negative Scans, *n* = 86
**Age at scan, median (range), y**	69.2 (50.9–80.5)	68.1 (52.3–80.5)	69.7 (50.9–79.2)
**PSA before treatment, median (range), ng/mL**	10.5 (0.9–183)	10.8 (3.2–183)	10.3 (0.9–138)
Unavailable (*n*)	5	3	2
**Initial therapy (*n*)**			
*RP*	153	68	85
*RT*	36	35	1

**Table 2:** Tumor characteristics during primary staging (All patients).

Characteristic	All Patients, *n* = 189	PET Positive Results, *n*, (%)	χ^2^
**PSA before treatment, ng/mL**			*p* = 0.948
<10	86	46 (53.5)	
10–20	57	32 (56.1)	
>20	41	22 (53.7)	
Unavailable (*n*)	5	-	
**cT-stage, prior treatment**			*p* = 0.667
<cT2a	70	35 (50)	
cT2b	10	6 (60)	
>cT2c	38	22 (57.9)	
Unknown	71	-	
**ISUP Grade Group, prior treatment**			*p* = 0.832
1	30	18 (60)	
2	58	30 (51.7)	
3	31	17 (54.8)	
4	45	26 (57.8)	
5	20	9 (45)	
Unknown	5	-	
**Risk stratification (D’Amico)**			*p* = 0.629
Low	18	9 (50)	
Intermediate	70	41 (58.6)	
High	95	49 (51.6)	
Unknown	6	-	

**Table 3:** Patients initially treated with RP.

	All RP Patients, *n* = 153	PET-Positive Results, *n*, (%)	χ^2^
**ISUP Grade Group, prostatectomy**			*p* = 0.504
1	10	7 (70)	
2	55	22 (40)	
3	38	18 (47.4)	
4	22	9 (40.9)	
5	23	10 (43.5)	
Unknown	5	-	
**Surgical margins**			*p* = 0.577
Positive	48	20 (41.7)	
Negative	101	47 (46.5)	
Unknown	4	-	
**pT-stage**			*p* = 0.173
<pT2a	5	1 (20)	
pT2b	2	2 (100)	
>pT2c	145	64 (44.1)	
Unknown	1	-	

**Table 4:** ^68^Ga-PSMA PET/CT detection rate dependent of PSA at recurrent disease, RP and RT combined.

Stratification	*n*	PET-Positive Results, *n*, (%)	χ^2^
All patients	189	103 (54.5)	*p* < 0.01
PSA ng/mL			
<0.5	82	23 (28)	
0.5 ≤ 1	23	9 (39)	
1 ≤ 2	25	16 (64)	
2 ≤ 5	24	21 (87.5)	
≥5	33	32 (97)	
Unknown	2	-	

**Table 5:** ^68^Ga-PSMA PET/CT detection rate dependent of PSA at recurrent disease, RP only.

Stratification	*n*	PET-Positive Results, *n*, (%)	χ^2^
All patients	153	68 (44.4)	*p* < 0.01
PSA ng/mL			
<0.5	79	21 (26.6)	
0.5 ≤ 1	22	8 (36.4)	
1 ≤ 2	23	14 (60.9)	
2 ≤ 5	12	9 (75)	
≥5	16	15 (93.8)	
Unknown	1	-	

## Data Availability

Most data supporting the reported results are provided in the paper and further data are stored by the authors.
